# Collectanea

**Published:** 1828-03

**Authors:** 


### COLLECTANEA.

Floriferis ut apes in saltibus omnia libant,
Omnia nos, itidem, depascimur aurea dicta.

### PHYSIOLOGY.

*Remarkable Hairy Man.*—As connected with this department, may be mentioned the existence at Ava of a man covered from head to foot with hair, whose history is not less remarkable than that of the celebrated porcupine man, who excited so much curiosity in England and other parts of Europe near a century ago. The hair on the face of this singular being, the ears included, is shaggy, and about eight inches long. On the breast and shoulders it is from four to five. It is singular that the teeth of this individual are defective in number, the molares, or grinders, being entirely wanting. This person is a native of the Shan country, or Lao, and from the banks of the upper portion of the Saluen, or Martaban river: he was presented to the king of Ava as a curiosity, by the prince of that country. At Ava he married a Burmese woman, by whom he has two daughters: the eldest resembles her mother; the youngest is covered with hair like her father, only that it is white or fair, whereas his is now brown or black, having, however, been fair when a child, like that of the infant. With the exceptions mentioned, both the father and his child are perfectly well formed, and indeed, for the Burman race, rather handsome. The whole family were sent by the king to the residence of the mission, where drawings and descriptions of them were taken.—(Jameson's *Journal*, 1827.)

### SURGERY.

*Case in which the Canal of the Larynx, after being nearly obliterated, was re-established.*—Elizabeth Oswald, æt. twenty-seven, in attempting suicide in September 1826, had wounded the larynx through the crico-thyroid ligament. She was under treatment for some months, and was at length abandoned with the loss of voice, and breathing entirely through a silver tube introduced in the situation of the original wound. On withdrawing this tube, and examining the parts attentively, a very minute orifice, capable of admitting the point of a probe of half the usual size, was discerned leading towards the glottis.

About April 15, 1827, Mr. Liston saw her by the request of Mr. Sidey, who had attended much to the case from the first, and began to introduce bougies about the size of darning needles into the part of the trachea above the wound; and, by gradually increasing the size of the bougies, the passage was brought, about the end of June, to its natural diameter. About the middle of August, the lower part of the trachea was dilated by a tube gradually increased in size, so that one of sufficient diameter might be passed by the mouth.

Three weeks afterwards the short tube was removed, and a long one was introduced by the wound up into the mouth, there laid hold of and pushed down into the trachea. This was followed by a very severe fit of coughing, which lasted about half an hour.

This tube, which was about nine inches long, and equal in diameter to that of the largest œsophagus tubes, was retained in the windpipe for fifteen days, during which it caused great salivation, which loosened the teeth and extremely reduced the strength of the patient. In consequence of this it was necessary to use some active means for the closure of the wound. On the fifth day the cicatrix was dissected out by two elliptical incisions, and the edges of the wound united by suture. The tube being removed on the fifteenth day, (October 5th,) she breathed well. In the morning, however, her breathing became very difficult, and tracheotomy was performed, and a silver tube introduced, which was kept in five days, and then replaced by a smaller one.

The actual cautery was applied to the edges of a small sinus, which remained on October 17th; and on the 26th, it was reapplied to the opening, which had very nearly closed. On the 28th, the tube was removed; and a few days after the wound was completely closed. The woman now breathes with comparative ease through the larynx, and is slowly recovering the use of her voice. (*Edinburgh Med. and Surg. Journal.*)

---

*Causes of the Fatal Termination of certain Cases of Bronchotomy,* by Dr. Cullen.—By the united researches of the French academicians and our own most intelligent surgeons, it was indisputably proved that bronchotomy afforded the only chance of relief in the case of foreign bodies in the trachea, and the only chance of recovery in many cases of cynanche laryngea.

It was proved also that wounds of the larynx or trachea, though inflicted on membranous or cartilaginous parts, were not, on that account merely, of a very dangerous nature, as the ancients had erroneously supposed; and the absolute safety of the operation was perhaps too confidently maintained. At least, I must confess that I have been induced to form an opposite opinion from reflecting deeply on the uses of the larynx, and on symptoms which I have witnessed in cases that have fallen under my observation.

In illustration of the opinion that wounds of the air-passages, even when inflicted on a healthy subject, are not only not trifling, but often highly dangerous, and followed by fatal consequences, the following case, which was treated by me in the Royal Infirmary, may be quoted.

A. B., a healthy-looking married woman, twenty-five years of age, and mother of one child, was subjected at home to many causes of mental irritation from the misconduct of her husband, and the unkindness of her friends. These acting on an irritable habit, produced a great depression of spirits, under the influence of which she tried to lay violent hands on herself, by cutting her throat with a table-knife. The instrument, however, was blunt, and she failed in her object, having been able to make only a slight incision, of about three inches in length, through the skin and cellular tissue over the crico-thyroid membrane. The latter likewise was divided to a small extent, and an opening was made into the larynx hardly capable of admitting the point of the little finger.

The accident occurred at ten A.M. When I saw the patient about noon, I found the wound in the situation, and of the size, above described; she breathed entirely through it, and consequently her voice was lost, except when the orifice was stopped up, when she spoke quite distinctly by means of the glottis. She had no difficulty of breathing, no attempt at coughing, no hemorrhage. In fact, she made no complaint. The case, however, seemed otherwise alarming. She was slightly delirious, always on the subject of her domestic grievance; the pulse was small and feeble; her face was rather flushed; the tongue was white and tremulous when protruded; and the countenance had a peculiar expression of anxiety and suffering. By means of a proper bandage, the head was bent down upon the sternum, so as to close the orifice in the crico-thyroid membrane, upon which a simple pledget was applied, no other dressing being thought necessary, as it was determined to let the wound heal slowly, in its own way, by granulation.

The head symptoms seemed much more urgent; inflammation of the arachnoid membrane was suspected, a disease which I have uniformly found most difficult of cure. When depending on mental distress, it is a complaint often fatal, particularly when, occurring in those who have attempted their lives, it is treated too actively by blood-letting. I contented myself, therefore, with desiring that the patient should have her head shaved, and that ice should be applied to it; that she should occasionally have a small quantity of wine-and-water, if she seemed disposed to sink; that a brisk cathartic should be given; and that she should be removed to a darkened chamber, where she could be kept cool and perfectly quiet.

These measures appeared to have a very salutary effect. At seven P.M. she was more composed; the expression of the countenance was improved; the dressings were displaced, and she breathed fully from the external orifice. The wine was ordered to be stopped, and the ice to be regularly continued.

The next morning, she was reported to have passed a quiet but sleepless night. The pulse was firmer than before. She had some cough, or rather violent expirations in place of cough, unaccompanied by sputum of any kind.

In the evening, the delirium seemed to have increased, with tendency to noise and violence. The respiratory symptoms were the same as before. Ten leeches were ordered to be applied to the forehead and temples.

Next morning, she was rather better; the cough, however, was somewhat increased, and she had slight expectoration of viscid clear transparent mucus. This expectoration was brought up with great difficulty. It was expelled through the wound, not by coughing, strictly so called, but by expiration merely; and that with so little force, that it had to be pulled out of the wound by the fingers of the patient or the nurse. A mucilaginous antimonial mixture was ordered.

In the evening, the symptoms were much the same as in the morning; the expectoration rather more copious; and the stethoscope detected acute inflammation in the smaller branches of the bronchi. A large blister was applied to the sternum, as the state of the intellectual functions and constitutional powers seemed to forbid the use of the lancet.

Next morning the patient was thrown into considerable agitation by a visit from her husband. She was very delirious at the visit at noon. The sputum was more copious, and still got rid of with much difficulty. She was ordered an anodyne draught, which procured a few hours' quiet repose, and soothed her excited imagination. I was in hopes, therefore, that the patient was to do well, as she had no threatening symptoms referable to the chest, and as the head symptoms were comparatively moderate and manageable.

Next morning, however, at five o'clock, Dr. Lubbock, the house-surgeon, to whom I am much indebted for his assistance in the management of the case, was suddenly roused from his bed, as the patient was reported to be dying. He found her on the point of suffocation, breathing very hurriedly with great difficulty, with the death rattle in her throat, livid face, and cold extremities. She was unable to expectorate. Dr. Lubbock immediately introduced a large curved canula into the wound, through which the patient was enabled to take a full inspiration. The effect of the introduction of the instrument was, that the breathing became much easier; and, as the alarming symptoms evidently arose from the accumulation of mucus in the bronchi, she was strongly recommended to cough as much as she could. By degrees she was enabled to bring up mucus through the canula, not, however, by coughing, but simply by expiration, which, as the canula was of large size, she could perform with considerable force.

In the course of a short time, as the expectoration became more free, the alarming symptoms subsided; the rattle in the throat was no longer heard, the breathing became easy, and the expression of the countenance very greatly improved.

Having been thus apparently rescued from the very jaws of death, she was left under the care of the nurse, who was directed to request her from time to time to make attempts at coughing. At noon, when I paid her a visit along with Dr. Lubbock, I found her very nearly in the same state from which she had been recovered with so much difficulty in the morning. The canula had slipped out of the trachea; the extremities were cold, the lips livid, the pulse hardly perceptible; the respirations rare, difficult, and accompanied with a frightful rattle in the bronchi; no expectoration; no attempt at coughing; and a great degree of stupor. In a loud tone of voice I desired her to cough, *but she could not make the attempt.* To enable her to expectorate, to allow her to cough, I placed my thumb on the orifice of the crico-thyroid membrane, so that she breathed entirely by the glottis much more freely than she had done before through the artificial opening.

When the respiration was a little improved, I requested her to cough. She could now do so readily enough, and brought up some very tenacious mucus, by the excretion of which the respiration was still further improved.

In the course of about a quarter of an hour, in consequence of the free expectoration, the breathing became quite easy; the expression of the countenance improved; and the lips regained nearly their natural hue. I directed a dresser to remain constantly with the patient, to keep the crico-thyroid membrane closed with his thumb, and to direct her to make attempts at coughing from time to time, and, if she refused, to tickle the epiglottis with a feather. For about an hour and a half the patient complied with the directions given her, and coughed up a considerable quantity of mucus; but by degrees she became more and more insensible, and would not cough; titillation of the epiglottis produced no effect; the mucus accumulated in the bronchi, and she died of suffocation about four o'clock.

*Sectio cadaveris, twenty-one hours after death.*—Upon opening the cranium, the vessels were found much more numerous than usual, more highly injected, especially on the surface of the right hemisphere. There was a good deal of serum in the subarachnoid cellular tissue, but the serous surface of the membrane was perfectly natural.

The substance of the brain was more vascular than usual, but not inflamed, softened, or otherwise disorganised. There was a considerable quantity of serum in the interior of the ventricles; a large quantity also at the basis of the brain, where likewise the membranes were much more vascular than usual, particularly on the tuber annulare.

Upon opening the chest, the pleura was found in a perfectly natural state.

The bronchi, particularly the left, were obstructed with bloody mucus. There was a small quantity in the trachea. The lungs were every where soft, crepitating, elastic, and only a little more vascular than usual.

In the neighbourhood of the wound, the mucous membrane of the larynx was inflamed, and red from increased vascularity, but it was not thickened. There was no effusion of fluid into the submucous cellular tissue, and consequently no diminution in the diameter of the glottis. Immediately at the edge of the wound, there was a slight effusion of lymph. The membrane of the trachea and that of the bronchi were inflamed and highly vascular, not thickened, but perhaps a little softer than natural; and it seemed as if the inflammation had spread down the air-passages, into the minute branches of the bronchi.

This case illustrates in a very forcible manner the truth of some observations on bronchotomy, published in the Number of this Journal for July last. I took occasion in that paper to call the attention of the profession to the physiology of coughing, and to the danger which necessarily attends on a copious secretion of mucus from the bronchi when an opening has been made in the trachea. For, to produce full and vigorous coughing, so as to clear the minute branches of the bronchi of their fluid secretion, the expiratory muscles must for a certain time act in opposition to the constrictor muscles of the glottis. When by this antagonism the former have acquired sufficient force, the latter are suddenly relaxed, and in the moment of their relaxation coughing takes place, and mucus or pus is expectorated along with the air thus violently expelled; but, when an opening is made into the trachea, expectoration immediately becomes difficult, for the expiratory muscles have no longer their antagonist constrictors of the glottis to act against. They cannot therefore acquire sufficient force to produce that strong current of air which is at least highly favorable to expectoration. A patient, therefore, after bronchotomy, cannot perform the act of coughing, properly so called. He can only expire with a force proportioned to the quantity of air he can take into the lungs.

The truth of these observations is borne out by what I have noticed in all the cases in which I have seen bronchotomy performed. I have uniformly observed that expectoration immediately became difficult after the operation. The patient could not drive the expectorated matter through the canula, for the current of air was too weak; and hence I have seen death ensue from a small quantity of blood getting into the trachea.

It was my thorough conviction of this truth that made me close the external wound entirely, when I found the patient on the point of suffocation. The effect of the closure was, that the muscles of the glottis were called into play; the patient was enabled to cough, and consequently to expectorate, so as to get rid of the mucus which obstructed the bronchi: she, however, had a few hours before derived equal relief from a directly opposite practice,—viz. the introduction of a canula into the wound; and the alarming symptoms had recurred only because the instrument had been displaced. This fact admits of a very satisfactory explanation. A full inspiration is favorable to expectoration, and the force of the expiratory muscles is in proportion to the quantity of air inhaled. The wound in the patient's throat, never very large, was considerably diminished by the swelling of the surrounding parts; she could not therefore take in a large quantity of air, while, on the other hand, she had lost the use of the constrictor muscles of the glottis; and therefore her expectoration was difficult, or rather impossible. But when a large canula was introduced, she could take in a large volume of air, on which the expiratory muscles could act with such force as to produce expectoration. Both practices, therefore, though diametrically opposed to each other, were perfectly judicious under the circumstances of the case; but on far different grounds. The closure of the external orifice allowed the muscles of the glottis to act, and genuine coughing to take place; by the introduction of a wide canula, a large quantity of air found admission to the lungs, which is to a certain degree a substitute for proper coughing.

These considerations lead to some important practical views, and enable us to explain the fatal termination of many of the recorded cases of bronchotomy. In the first place, the canula employed ought always to be large. If it be small, the breathing will be difficult. During the inspiration, blood will be sucked into the trachea from the edges of the wound, and a quantity of air sufficient for the purposes of respiration will not be admitted to the lungs. Secondly, when inflammation exists in the minute branches of the bronchi, attended with plentiful secretion, the introduction of a large quantity of air is absolutely necessary for expectoration. A small quantity of air cannot acquire sufficient force to bring up along with it the secretions of the bronchi. We can explain on the same principle why bronchotomy is so rarely successful when diseases have extended into the minute branches of the bronchi. After the operation the expectoration becomes more difficult, from the constrictor muscles of the glottis being rendered useless. Mucus accumulates more and more down the air-passages, and the patient dies gradually suffocated.

In severe cases of inflammation of the lungs, the expectoration is commonly suppressed a short time previous to death, but it does not cease to be secreted, it is only not excreted; for, in proportion as more and more of the lungs becomes useless for the purposes of respiration, of course a less quantity of air can be introduced at each inspiration, till at last the quantity is insufficient for a forcible inspiration or coughing; then, of course, the minute branches of the bronchi continue loaded, and the patient is suffocated. These observations on the influence of bronchotomy on coughing and expectoration were partially laid before the profession in the Number of this Journal for July last; but the paper was too concise, and was misunderstood, particularly by the editor of the London Medico-Chirurgical Review. That gentleman may depend upon it that it is not wrong to say that a patient with a tube in the trachea can most commonly only produce a current of air too weak for the purposes of expectoration. I have never seen a patient expectorate matter through the canula with any thing like freedom. It has been always necessary to extract the tenacious mucus by probes and other mechanical means. There has been uniformly a want of expiratory power, which the constrictors of the glottis alone could have supplied. My professional friends have assured me that their experience has been of the same kind: they have witnessed the same difficulty of expectoration. The editor, likewise, as an argument against the truth of my views, objects that, in the case formerly published, I had distinctly stated that, on the first introduction of the tube, a fit of coughing supervened, attended, to my great surprise, with expectoration of semitransparent viscid mucus. I was certainly surprised, because I had no reason to believe that any mucus was secreted by the bronchi; but the expectoration of it immediately after the introduction of the tube is not only not in opposition, but very strictly in accordance with my views of the physiology of the windpipe. The patient had laboured under laryngitis, by which the size of the rima glottidis had been so far diminished that a very small quantity of air could be introduced at each inspiration,—too little, indeed, for proper coughing. But as soon as an opening was made in the crico-thyroid membrane, and a large canula introduced, then the patient could breathe freely, and, though she could not cough, properly speaking, she could expire with considerable force, and thus get rid of a quantity of mucus in the way of expectoration.

From the foregoing statements I would draw the following practical conclusions:—

1st. Bronchotomy should not be performed in cases where inflammation and copious secretion exist in the minute branches of the bronchi,—at least such cases are most likely to terminate unfavorably. The operation, if performed, may relieve the breathing for a while, but it may occasion in a short time a suppression of the expectoration.

2d. We should always make use of a large canula for the sake of permitting free expiration, the only substitute for coughing, which the patient can no longer effect.

3d. The operation of bronchotomy is not in itself unattended with danger; and even slight wounds of the trachea may be fatal from their effect in suppressing the excretion of the mucus of the air-passages. The case which I have just related is a good illustration of the manner in which a slight wound of the trachea may be fatal. The patient, a woman in the vigour of life, most carefully watched and attended to, died in consequence of a slight wound of the crico-thyroid membrane, because the inflammation extended down the windpipe into the bronchi, and was attended with secretion, which could not be expectorated on account of the wound below the glottis.

The same fatal termination had very nearly occurred in a young woman in the Royal Infirmary, a patient of my colleague Dr. Ballingall, during the autumn of last year. She, too, had cut her throat slightly after her delivery of a still-born natural child. The wound was comparatively slight, and required no peculiar treatment; but the inflammation had extended down into the minute branches of the bronchi, and was attended with copious secretion. While the wound was closing, the expectoration became so difficult that suffocation was repeatedly threatened, which was averted, however, by the enlargement of the wound, and the introduction of a large canula.

I remember to have surprised the house-surgeon, Dr. Lubbock, by stating that equal relief would have followed the complete closure of the wound by the thumb or forefinger.

4th. The absence of expectoration may be either very favorable or unfavorable to the performance of bronchotomy. It is a very favorable symptom when it depends on the sound state of the mucous membrane of the bronchi. It is most unfavorable when it depends on such a closure of the rima glottidis that too small a quantity of air can be introduced to effect coughing.—(Dr. Cullen, *Ibid.*)

---

*Stricture of the Lachrymal Duct.*—The following is Dupuytren's method of treating this affection:—

The instruments employed in the operation are a common French bistoury, a canula of silver, gold, or platina, and a *porte-canule*, to which the canula is affixed for being more conveniently inserted in the lachrymal passage. The length of the canula is one inch; its upper opening measures one-tenth of an inch; the tube gradually tapers from its upper to its lower opening, the diameter of which measures one-twentieth part of an inch; the lower opening is placed obliquely, and the upper opening is surrounded by a small rim, to prevent its sinking too low into the duct.

The following is the manner in which the operation is performed:—The patient being seated in a chair, rests the head against the breast of an assistant, who fixes the head by a hand placed over the ear and temple on each side. The operator (supposing the left eye to be operated upon) stands in front of the patient, and a little to the left side. With the index finger of the left hand he puts the integuments at the inner angle of the eye upon the stretch, for the purpose of rendering the tendon of the orbicularis palpebrarum more evident, at the same time that the assistant, who holds the head, stretches the integuments at the outer angle of the eye. The lachrymal sac is then to be punctured by the point of the bistoury being inserted immediately below the tendon of the orbicularis palpebrarum. The direction in which the instrument should be inserted is nearly vertical, the handle inclining slightly to the opposite side, and a little forwards, on account of the projection of the eyebrow, the edge of the blade being directed obliquely downwards, or towards a point situated midway between the angle of the mouth and the pinna of the ear, this being the direction which admits of the bistoury being insinuated to the greatest extent down the lachrymal canal. The puncture being made, the bistoury is allowed to remain as a director, and the operator, taking in the left hand the porte-canule armed with the canula, gently insinuates the tube along the side of the bistoury into the lachrymal canal; the bistoury is then withdrawn; slight pressure is made upon the upper part of the canula, so as to cause it to be completely imbedded in the canal. The operation being now completed, the patient is directed to blow through the nose at the time that pressure is made upon the nostrils, by which the tube is freed from any blood that may have accumulated in it, and the air issues out at the incision, showing the free communication that is established between the nostril and the sac.

---

### PHARMACY.

*Volatile Oil of Copaiba.*—We have lately seen some volatile oil obtained by distillation from copaiba, by Mr. Battley, of Fore-street. It agrees in its physical and chemical properties with the oil of turpentine, excepting in its smell, which is somewhat different. There can be but little doubt that on this oil the powers of copaiba as a remedial agent depend; and, if this be the case, the resin of copaiba, concerning which so much has been lately said, can have little or no activity, in consequence of this oil having been driven off. Several trials with the resin have proved the correctness of this inference. The volatile oil of copaiba requires a heat of about 600° F. to obtain it, and is then procured only in a very small quantity.

---

*Preparation of Sulphate of Quinia and Kinic Acid, without the use of Alcohol.*—The following is the process of MM. Henry and Plisson: About two pounds of bark are to be coarsely powdered, and boiled with water, acidulated with sulphuric acid in the usual manner. When the hot liquors are cleared, recently prepared and moist hydrate of lead is to be added until the fluid is neutral, and has acquired a faint yellow colour: this must be done carefully, lest too much hydrate of lead be added. As the decoloration of the decoction is necessary, the liquid, if it remains turbid until the next morning, must have a little more hydrate added, and be refiltered; but the operation is rarely subject to this inconvenience, being usually finished in a few hours. The yellow liquid contains a little kinate of lead, much kinate of lime, kinate of quinia or cinchonia, a little colouring matter, and traces of other substances. The washed deposite consists of colouring matter, combined with oxide of lead, sulphate of lead, and a portion of free quinia; it contains no sub-kinate of lead.

The lead, dissolved in the fluid, is to be separated by a few drops of sulphuric acid, or a small current of sulphuretted hydrogen, and the filtered liquid is to be precipitated by adding caustic lime, previously mixed into a thin paste with water, until the earth is in very slight excess: in this manner the quinia is precipitated. The addition of sulphuric acid readily converts this quinia into sulphate, which may be obtained in very white and silky crystals. The fluid left after the separation of the quinia contains a kinate of lime, almost pure. Being evaporated until of the consistence of syrup, it readily crystallizes in a mass, which may then be purified by recrystallization. The kinate of lime may be precipitated by means of alcohol, and then be crystallized after solution in water or diluted alcohol; or, by adding oxalic acid drop by drop, according to the directions of M. Vauquelin, the lime may be separated, and kinic acid obtained. Two-thirds of the quinia or cinchonia in a specimen of bark may be thus separated, and with such facility as to offer a ready test of the presence of these alkalies in any wood or bark submitted to examination. (*Journal of Science*, from the *Ann. de Chimie.*)

---

*Pure Narcotine.*—The following process is that practised by Mr. Carpenter: Digest one ounce of coarsely powdered opium in one pint of ether for ten days, frequently submitting it to ebullition in a water bath; separate the ether, and add fresh portions until the opium is exhausted; place the ethereal solution in a wide-mouthed bottle, and, covering the mouth with bibulous paper, allow the ether to evaporate spontaneously, but slowly: as the fluid diminishes, it leaves the sides of the bottle coated with crystals of narcotine; as the solution becomes more dense, the crystals enlarge and accumulate, and the bottom of the vessel is covered with large transparent crystals, accompanied with a brown viscid liquor and extract, which contains an acid resin, caoutchouc, &c. Separate these substances, and wash the crystals in successive portions of cold ether, to remove the extract; then dissolve them in warm ether, and evaporate slowly as before: beautiful snow-white crystals of pure narcotine will be obtained: those on the sides of the vessel assume plumose and arborescent forms; they enlarge as the solution becomes more concentrated, and the bottom of the bottle becomes covered with pure narcotine, assuming the rhomboidal prismatic form, with some modifications of maccled crystals. The crystals towards the bottom are transparent, but the most minute at the top are opaque and snow white. By picking out the largest and most regular crystals, again dissolving and evaporating, and repeating the same process, each time selecting the largest and best crystals, some were obtained the eighth of an inch in diameter, and still larger might be produced by similar operations. (Silliman's *Journal.*)

